# Carboplatin–paclitaxel-induced leukopenia and neuropathy predict progression-free survival in recurrent ovarian cancer

**DOI:** 10.1038/bjc.2011.256

**Published:** 2011-07-12

**Authors:** C K Lee, H Gurney, C Brown, R Sorio, N Donadello, G Tulunay, W Meier, M Bacon, J Maenpaa, E Petru, N Reed, V Gebski, E Pujade-Lauraine, S Lord, R J Simes, M Friedlander

**Affiliations:** 1 NHMRC Clinical Trials Centre, University of Sydney, Locked Bag 77, Camperdown, NSW 1450, Australia; 2 Department of Medical Oncology, Westmead Hospital and Department of Medicine, University of Sydney, Sydney, NSW 2774, Australia; 3 CRO Aviano, via Franco Gallini, 2 33081 Aviano (PN), Italy; 4 Clinica Ostetrica Ginecologica, Ospedale F. Del Ponte, Via F. Del Ponte 19, 21100 Varese, Italy; 5 Etlik Zubeyde Hanim Women′s Teaching and Research Hospital Gynecologic Oncology Clinic, Ankara, Turkey; 6 Department of Gynecology and Obstetrics, Ev. Hospital, Duesseldorf, Germany; 7 Secretariat, Executive Board, Study Coordinator, GCIG Secretariat, Queen′s University—Cancer Research Institute, NCIC Clinical Trials Group, 10 Stuart Street, Kingston, ON K7L 3N6, Canada; 8 Department of OB and GYN, Tampere University Hospital, PO Box 2000, FI-33521 Tampere, Finland; 9 Universitätsklinik für Frauenheilkunde und Geburtshilfe der Med. Universität Graz, Graz, Austria; 10 Beatson Oncology Centre, Gartnavel General Hospital, 1053 Great Western Road, GLASGOW, G12 0YN Scotland, UK; 11 Université Paris Descartes, AP-HP, Hôpitaux Universitaires Paris Centre, Site Hôtel-Dieu, Oncologie, 1 Place du Parvis Notre-Dame, 75004 Paris, France; 12 Prince of Wales Hospital, Institute of Oncology, High Street, Randwick, Sydney, NSW 2031, Australia

**Keywords:** leukopenia, neuropathy, ovarian cancer, prognosis, prediction

## Abstract

**Background:**

We assess the prognostic value of chemotherapy-induced leukopenia and sensory neuropathy in the CALYPSO trial patients treated with carboplatin–paclitaxel (CP) or carboplatin–liposomal doxorubicin (CPLD).

**Methods:**

We performed a landmark analysis at first month after randomisation to correlate leukopenia (nadir white blood cell <4.0 × 10^9^ per litre or severe infection) during cycle 1 of chemotherapy with progression-free survival (PFS). Using time-dependent proportional-hazards models, we also investigated the association between neuropathy and PFS.

**Results:**

Of 608 patients with nadir blood and did not receive growth factors, 72% (CP=70%, CPLD=73%) had leukopenia. Leukopenia was prognostic for PFS in those receiving CP (adjusted hazard ratio (aHR) 0.66, *P*=0.01). Carboplatin–liposomal doxorubicin was more effective than CP in patients without leukopenia (aHR 0.51, *P*=0.001), but not those experiencing leukopenia (aHR 0.93, *P*=0.54; interaction *P*=0.008).

Of 949 patients, 32% (C*P*=62%, CPLD=28%) reported neuropathy during landmark. Neuropathy was prognostic for PFS in the CP group only (aHR 0.77, *P*=0.02). Carboplatin–liposomal doxorubicin appeared to be more effective than CP among patients without neuropathy (aHR 0.70, *P*<0.0001), but not those with neuropathy (aHR 0.96, *P*=0.81; interaction *P*=0.15).

**Conclusion:**

First-cycle leukopenia and neuropathy were prognostic for patients treated with CP. Efficacy of CP treatment was similar to CPLD in patients who developed leukopenia. These findings support further research to understand the mechanisms of treatment-related toxicity.

Combination chemotherapy with carboplatin and paclitaxel (CP) has been the standard of care for patients with platinum-sensitive recurrent ovarian cancer. Treatment with CP is associated with longer progression-free survival (PFS) and overall survival than conventional platinum-based chemotherapy (Parmar *et al*, 2003; Pfisterer *et al*, 2006). More recently, the CALYPSO study has demonstrated that carboplatin and liposomal doxorubicin (CPLD) is associated with less toxicity and improved PFS compared with CP using standard doses (Pujade-Lauraine *et al*, 2010).

However, more than half of these patients relapse within a year. In addition to finding novel therapies, further research is required to identify factors associated with benefit using available chemotherapeutic agents, which may allow individualisation of treatment and improve efficacy.

Leukopenia and sensory neuropathy are common toxicities of paclitaxel (Rowinsky *et al*, 1993a,1993b). These toxicities may reflect both the level of exposure and tissue susceptibility to this chemotherapy. Several studies have demonstrated that myelosuppression is associated with improved clinical outcomes in a number of cancers treated with various chemotherapeutic agents (Saarto *et al*, 1997; Poikonen *et al*, 1999; Di Maio *et al*, 2005; Yamanaka *et al*, 2007; Koutras *et al*, 2008; Pallis *et al*, 2008; Rocconi *et al*, 2008; Shitara *et al*, 2009, 2010b). An association between sensory neuropathy and clinical outcome has not yet been established.

We hypothesised that in patients with platinum-sensitive ovarian cancer treated with CP, leukopenia and sensory neuropathy would be associated with superior PFS and used data from the CALYPSO trial to test this hypothesis. We also investigated whether these particular toxicities predicted clinical efficacy in patients treated with CP compared with those treated with CPLD.

## Materials and methods

### Patient population

The CALYPSO study has been reported (Pujade-Lauraine *et al*, 2010). The primary objective of this non-inferiority phase III trial was to evaluate the safety and efficacy of the CPLD administered at 4-weekly intervals compared with CP at 3-weekly intervals on PFS. Eligible patients were enrolled between April 2005 and September 2007. Patients were evaluated for tumour response or progression every 3 months while on chemotherapy. In both arms, six cycles of treatment in the absence of progressive disease or unacceptable toxicity were planned; continuation beyond six cycles was at the discretion of the treating clinicians.

### Chemotherapy dosing and use of granulocyte-colony stimulating factor

In the first cycle, patients randomised to CPLD received PLD at 30 mg m^*−*2^ intravenously on day 1 and carboplatin at area under the time-concentration curve (AUC) 5, based on the Calvert formula, using the glomerular filtration rate (GFR) calculated from serum creatinine values according to the method of Cockroft and Gault, and administered intravenously on day 1 at 4-week intervals. Patients randomised to the CP arm received paclitaxel at 175 mg m^*−*2^ intravenously on day 1 and carboplatin at AUC 5 intravenously on day 1 at 3-week intervals. Dose escalation of carboplatin to AUC 6 in the absence of significant toxicity was allowed. Dose reduction due to excessive toxicity was allowed but dose re-escalation in the subsequent cycles was prohibited.

Prophylactic treatment with granulocyte-colony stimulating factor (G-CSF) was allowed and optional. Supportive treatment with G-CSF was allowed if patients developed neutropenia (absolute neutrophil count nadir <0.5 × 10^9^ per litre lasting for >5 days), febrile neutropenia or sepsis in any cycle of chemotherapy.

### Leukopenia and sensory neuropathy evaluation

Blood tests were performed within 2 weeks of study enrolment, at mid-cycle of chemotherapy and up to 3 days before the next cycle of chemotherapy. The study protocol, however, did not require mandatory evaluation of nadir blood count.

Baseline evaluations for sensory neuropathy were performed within 2 weeks of study enrolment. Clinical re-evaluations for sensory neuropathy were performed up to 3 days prior each new cycle of chemotherapy.

### Leukopenia and sensory neuropathy definitions

Patients were classified as leukopenic, based on nadir count of first chemotherapy cycle, with white blood cells (WBCs) <4.0 × 10^9^ per litre (NCI-CTCAE grade ⩾1). Patients were also classified as leukopenic, regardless of the nadir count of the first chemotherapy cycle, if they developed febrile neutropenia or severe infection (NCI-CTCAE grade ⩾3) after the first cycle of chemotherapy. Patients who were treated with G-CSF starting from the second cycle, regardless of the nadir count of first chemotherapy cycle, were also classified as leukopenic. Patients were excluded if G-CSF was used prophylactically as part of the first cycle, or if they progressed or died within the first month of treatment.

Sensory neuropathy was defined as new onset of neuropathy (NCI-CTCAE grade ⩾1) or worsening of pre-existing neuropathy graded according to NCI-CTCAE criteria during treatment. Patients were excluded if there was no assessment of neuropathy at baseline and were required to have at least one assessment during treatment.

### Statistical methods

Baseline patient demographic and disease characteristics were compared by using *t*-tests for continuous variables and *χ*^2^-tests for categorical variables. We performed landmark analysis using a cutoff time of 30 days after randomisation and restrict the analyses to patients who were still alive and not progressed during these 30 days. The PFS_L_ is defined as time from landmark to documented evidence of disease progression by RECIST criteria, the occurrence of new disease or death from any cause. In patients who received study treatment without a progression date or death, PFS_L_ will be censored on the date of last clinical assessment. The PFS_L_ was estimated with the Kaplan–Meier method, and compared by leukopenic status, overall and by assigned treatment, using the log-rank tests. Proportional-hazards models were constructed to report hazard ratios (HRs) for PFS_L_ adjusting for baseline prognostic factors (treatment-free interval, presence of measurable disease, baseline CA-125 and baseline white cell count). A test of interaction between leukopenic status and treatment effect was also used to assess whether leukopenia predicted treatment effect.

In multivariable analyses, we modelled sensory neuropathy as a time-varying covariate over the entire course of treatment to examine whether it was prognostic of PFS. The PFS is defined as time from randomisation to documented evidence of disease progression by RECIST criteria, the occurrence of new disease or death from any cause. A test of interaction between neuropathy status and treatment effect was also used to assess whether neuropathy predicted treatment effect.

Exploratory analysis was also performed to test for an association between grade of leukopenia and PFS. Logistic regression models were constructed to characterise the relationship between leukopenia and neuropathy.

## Results

Of 975 trial patients, 608 (62%) (299 from the CP group and 309 from the CPLD group) received chemotherapy, did not progress or die during the first month of chemotherapy, did not receive prophylactic G-CSF and had a nadir blood count during cycle 1 of chemotherapy. Of these, 72% of patients (CP 70%, CPLD 73%) were classified as leukopenic. Table 1 summarises the patients’ baseline demographic and disease characteristics. Apart from baseline WBC levels, patients with and without leukopenia were similar.

### Prognostic and predictive value of leukopenia

**Figure 1:**
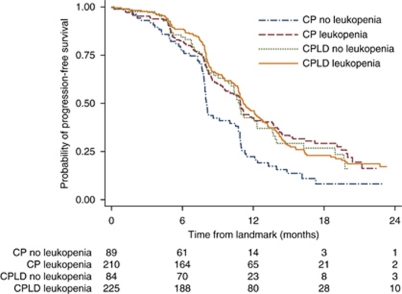
Kaplan–Meier survival in patients with and without leukopenia in the carboplatin–paclitaxel (CP) treatment group and the carboplatin–liposomal doxorubicin (CPLD) group. For the effect of leukopenia on PFS, in the CP arm, log-rank *P*=0.0009; in the CPLD arm, log-rank *P*=0.73; overall log-rank *P*=0.002.

In the overall population, the median PFS_L_ was 11.0 months for patients with leukopenia and 10.2 months for those without (HR=0.76; 95% CI=0.61–0.94; *P*=0.01). When examined according to treatment groups, leukopenia predicted better PFS_L_ in patients treated with CP (HR=0.61; 95% CI=0.45–0.82; *P*=0.001) but not with CPLD (HR=0.95; 95% CI=0.70–1.29; *P*=0.73; Figure 1). After adjustment for baseline prognostic factors, leukopenia remained significant in patients treated with CP (adjusted HR (aHR)=0.66; 95% CI=0.49–0.90; *P*=0.01) but not in CPLD.

### Severity of leukopenia and PFS outcome

**Figure 2:**
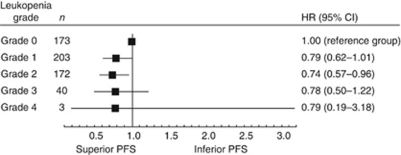
Prognostic value of leukopenia, by grade, for progression-free survival (PFS).

Among those with leukopenia, grades 1, 2, 3 and 4 leukopenia were detected in 49, 41, 10 and <1%, respectively. The PFS_L_ benefit increased with increasing severity of leukopenia (Figure 2; *P*_trend_=0.04).

### Treatment effect and leukopenia

**Figure 3:**
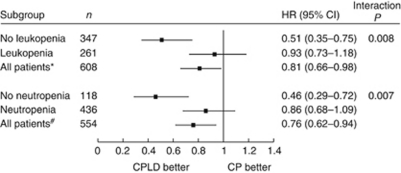
Effects of treatment on progression-free survival (PFS), after adjustment for baseline prognostic factors, for patients with and without leukopenia. CP, carboplatin–paclitaxel treatment group; CPLD, carboplatin–liposomal doxorubicin treatment group. ^*^Total patients with evaluable white cell count. ^#^Total patients with evaluable actual neutrophil count.

The treatment benefit of CPLD over CP was greater in patients without leukopenia (aHR=0.51; 95% CI=0.35–0.75; *P*=0.001), but this difference was not observed in those with leukopenia (aHR=0.93; 95% CI=0.73–1.18; *P*=0.54). The test of interaction between leukopenic status and the relative treatment effect of CPLD *vs* CP was significant (*P*=0.008; Figure 3).

Less data were available on neutropenia (absolute neutrophil counts ⩽2.0 × 10^9^ per litre), but similar effect was observed as leukopenia when the above analyses were repeated (Figure 3).

### Incidence of sensory neuropathy

Of the 975 patients, 949 (494 from the CP group and 455 from the CPLD group), received chemotherapy, had an assessment of neuropathy at baseline, and had at least one assessment of neuropathy during treatment. The mean number of neuropathy evaluations for patients across the entire study period was 5.6 (range 1–12) and 5.9 (range 1–14) in the CP and CPLD groups, respectively. One month after randomisation, development or worsening of sensory neuropathy was reported for 305 patients (61.7%) from the CP group and 125 (27.5%) from the CPLD group.

### Prognostic and predictive value of sensory neuropathy

**Figure 4:**
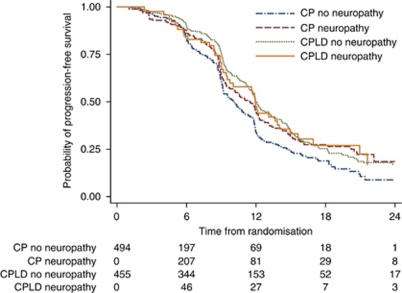
Kaplan–Meier survival in patients with and without sensory neuropathy in the carboplatin–paclitaxel (CP) treatment group and the carboplatin–liposomal doxorubicin (CPLD) group. For the effect of neuropathy, in the CP arm, log-rank *P*=0.02; in the CPLD arm, log-rank *P*=0.97; overall log-rank *P*=0.003.

There was significant difference in PFS between patients with and without neuropathy in the CP group (median PFS=11.5 *vs* 10.1 months; HR=0.77; 95% CI=0.62–0.95; *P*=0.02; Figure 4). Adjustment of baseline prognostic factors did not change this result (aHR=0.77; 95% CI=0.62–0.95; *P*=0.02).

In the CPLD group, there was no significant difference in PFS between patients with and without neuropathy (median PFS=12.1 *vs* 11.9 months; HR=1.01; 95% CI=0.72–1.40; *P*=0.97).

### Treatment effect and sensory neuropathy

The benefit of CPLD over CP appeared to be greater among patients without neuropathy (HR=0.70; 95% CI=0.58–0.84; *P*<0.0001), but this difference was not observed in those with neuropathy (HR=0.96; 95% CI=0.67–1.36; *P*=0.81). However, the test of interaction between neuropathy status and the relative treatment effect of CPLD *vs* CP was not significant (*P*=0.15).

### Relationship between leukopenia and sensory neuropathy

There was no relationship between leukopenia at landmark and development or worsening of sensory neuropathy from landmark (odds ratio (OR) (neuropathy *vs* no neuropathy)=0.91; 95% CI=0.64–1.29; *P*=0.59). However, for patients treated with CP who had NCI-CTCAE grade ⩾2 leukopenia (WBC <3.0 × 10^9^ per litre) at landmark, there was a non-statistically significant trend in development of neuropathy during the course of treatment (OR=1.53; 95% CI=0.94–2.49; *P*=0.08).

## Discussion

In this analysis, chemotherapy-induced leukopenia and sensory neuropathy were associated with improved prognosis in patients with platinum-sensitive recurrent ovarian cancer treated with CP. Women developing leukopenia during cycle 1 of CP had a 34% reduction in the risk of disease progression compared with those without leukopenia, with increasing severity of leukopenia associated with longer PFS. Likewise, development or worsening of sensory neuropathy on CP was associated with a 24% reduction in the risk of disease progression. Chemotherapy-induced leukopenia and sensory neuropathy were not prognostic for patients treated with CPLD. The CALYPSO trial reported superiority in PFS and better therapeutic index for CPLD over CP (Pujade-Lauraine *et al*, 2010). In this analysis, the treatment benefit was similar in patients treated with CPLD or CP only when CP induced leukopenia or sensory neuropathy, although a statistically significant interaction was only observed for leukopenia only (interaction *P*=0.008).

Our findings are consistent with a growing number of studies that report an association between chemotherapy-induced myelotoxicity and patient outcomes in a number of malignancies (Saarto *et al*, 1997; Poikonen *et al*, 1999; Di Maio *et al*, 2005; Yamanaka *et al*, 2007; Koutras *et al*, 2008; Pallis *et al*, 2008; Rocconi *et al*, 2008; Shitara *et al*, 2009, 2010b). A meta-analysis of 13 trials of various cancers (*n*=9528) reported a 31% reduction in risk of death for patients who developed grades 3 and 4 neutropenia or leukopenia compared with those experiencing lower grade or no cytopenia (Shitara *et al*, 2010a). In ovarian cancer, Rocconi *et al* (2008) reported improvement in PFS in patients who developed myelosuppression from platinum- and taxane-based chemotherapy. In another retrospective cohort study, Kim *et al* (2010) reported a non-significant increase in PFS and overall survival for patients with grades 3 and 4 neutropenia from treatment with CP compared with those with lower grade or no neutropenia. However, the multivariate analysis indicated that stage of disease, clear histology type and amount of residual disease were significant predictors of survival, but not neutropenia. In addition to the significant limitation of the smaller sample size to detect a true effect (*n*=130), several differences between the patient population and analytic techniques used in this study and the present analysis may have contributed to these conflicting results.

Lack of treatment-induced toxicity as surrogate for poorer patient outcome in the CP arm may be due to one of the several explanations. The first is that patients with less toxicity may have insufficient drug exposure and thus less cytotoxicity. Joerger *et al* (2007) demonstrated that using body surface area (BSA)-derived dose of paclitaxel led to a significant interpatient variation in drug exposure of >35%. This finding and others suggest that standard dose calculation methods may result in a significant proportion of patients being exposed to suboptimal drug concentration (Gurney, 2005). This may relate to variations in hepatic metabolism where polymorphism of P450 influences paclitaxel metabolism (Sonnichsen *et al*, 1995), whereas PLD is protected from hepatic metabolism (Hilmer *et al*, 2004). However, in our study, chemotherapy-induced leukopenia was prognostic of PFS only in patients treated with CP, indicating that myelotoxicity may be more reflective of adequate drug exposure in CP but not in CPLD. Leukopenia was a dose-limiting toxicity for paclitaxel in phase 1 trials (Rowinsky *et al*, 1993b). Unlike doxorubicin, PLD causes minimal myelosuppression due to a slower clearance rate and a longer elimination half-life (Hilmer *et al*, 2004). Instead, hand–foot syndrome and stomatitis are the dose-limiting side-effects of PLD (Uziely *et al*, 1995; Gordon *et al*, 2001). We did not observe a statistically significant association between hand–foot syndrome or stomatitis and PFS in patients assigned to CPLD in the CALYPSO trial (data not shown), suggesting that they may not be correlated with cytotoxicity in ovarian cancers. Alternatively, this lack of correlation may possibly be due to the limitations in the current grading system used to score toxicities.

The second explanation of the association between treatment-induced toxicity and anticancer effect observed in the CP arm may relate to genetic similarity between normal and malignant cells, resulting in both having similar susceptibility to the cytotoxic effects of the chemotherapy with adequate drug exposure. A number of genes have been proposed to be involved with drug resistance in ovarian cancer including the P-glycoprotein efflux transporter (PGP), anti-apoptotic proteins and other survival pathways (Richardson and Kaye, 2005). It is possible that, in any patient, the normal and malignant cells may share the same resistance mechanisms. For example, PGP, encoded for by *ABCB1* (also known as multidrug resistance gene 1), is affected by gene polymorphisms in tumour and non-malignant tissue. With taxane-based chemotherapy, the 2677G>T/A polymorphism of *ABCB1* is associated with leukopenia (Sissung *et al*, 2006; Tran *et al*, 2006), greater tumour response(Green *et al*, 2006), longer PFS (Johnatty *et al*, 2008) but higher drug clearance(Gréen *et al*, 2009). The 3435C>T polymorphism is associated with leukopenia (Tran *et al*, 2006) and neuropathy (Sissung *et al*, 2006) but not response (Green *et al*, 2006) or survival (Johnatty *et al*, 2008). However, these associations have not yet been validated, with small studies with different population of racial distribution reporting conflicting results. These studies have also not separated the clinical outcomes according to the polymorphisms occurring in the tumour (affecting resistance) or in normal tissue (affecting drug elimination) or both (Marsh *et al*, 2006, 2007). Such studies are required to determine whether these associations are due to issues related to drug exposure or shared drug resistance mechanisms between normal and tumour cells.

The findings of this study support further research into toxicity-adjusted dosing as a strategy to personalise treatment with recurrent ovarian cancer (Gurney, 1996). In general, this involves assigning an arbitrary toxicity target to infer that an adequate dose of chemotherapy has been given. Such research has been conducted in various cancers with variable results. In a large randomised phase III study of adjuvant chemotherapy for high-risk breast cancer patients, dose escalation based on lack of haematological toxicity resulted in 9% improvement in 3-year relapse-free survival (Bergh *et al*, 2000). In that study, the toxicity-adjusted dose led to a 3- to 4-fold range in doses of epirubicin and cyclophosphamide which is similar to the known interpatient variation in clearance of these drugs, as opposed to the <2-fold dose range achieved with BSA dose. However, the SCOTROC4 study of single agent carboplatin in advanced ovarian cancer failed to show a PFS benefit for patients who had dose escalation based on lack of neutropenia (Kaye *et al*, 2009). Carboplatin dose was derived using GFR which gives a narrow interpatient variation in drug exposure and leads to relatively less inadvertent under-dosing compared with BSA-derived dose. This is reflected in the relatively small difference in the dose of carboplatin received between the standard and the toxicity-adjusted arms (AUC 6.0 *vs* 6.8). As this study is yet unpublished, it will be important to review the range of doses received between the two arms (rather than just the median) with a particular focus on any overlap in the low-dose range.

A major strength of this study is the availability of high-quality large sample trial data from CALYPSO to investigate the study hypothesis. The assessment of toxicity and disease progression events was specified in the trial protocol according to standard definitions. Our analysis plan was designed to overcome the challenges for measuring the association between toxicity and disease progression. First, we have applied a landmark strategy by restricting the analysis to patients who had completed one cycle of chemotherapy and who were alive at 4 weeks after randomisation to minimise the potential for confounding by number of cycles of chemotherapy received as an explanation for the association observed between leukopenia and improved prognosis. A higher incidence of leukopenia would be expected as the number of cycles of chemotherapy increased due to cumulative bone marrow suppression, and patients with longer PFS would have receive additional cycles. Second, due to the cumulative effect of chemotherapy on development or worsening of sensory neuropathy, we performed a separate analysis where this toxicity is modelled as a time-varying covariate. This approach used all clinical assessments of neuropathy throughout the trial to provide a greater power to detect its prognostic value.

This study has a number of limitations. Approximately one third of the randomised patients did not have nadir blood count recorded during the first cycle of chemotherapy and were excluded from analysis. As compared with patients in this analysis, these patients without nadir blood count were different in terms of some of the baseline characteristics and other toxicities experienced during cycle 1 of chemotherapy (Table 1). However, there was no statistically significant difference in treatment effect (no nadir count, HR (CP *vs* CPLD)=0.79; 95% CI=0.60–1.04 and nadir count, HR (CP *vs* CPLD)=0.87; 95% CI=0.72–1.06; interaction *P*=0.46) or PFS in those with or without nadir blood count (10.9 *vs* 9.8 months, *P*=0.15). We have also adjusted for these baseline characteristics in our multivariate models.

In summary, first-cycle leukopenia and sensory neuropathy were associated with better prognosis in patients treated with CP, but not with CPLD in the CALYPSO trial. Efficacy of CP treatment was similar to CPLD in patients who developed leukopenia. These findings are hypothesis generating and should be interpreted with appropriate caution. However, these findings support further research to understand the mechanisms of treatment-related toxicity. Individualisation of chemotherapy dose based on toxicity should be explored in future trials.

## Figures and Tables

**Table 1:** Baseline characteristics of CALYPSO trial patients

	**No leukopenia (*n*=173)**		**Leukopenia (*n*=435)**		**No nadir count (*n*=311)**		
**Characteristic**	***N***	**%**	***N***	**%**	***N***	**%**	***P* a**
Median age, years	59.4		61.0		61.1		0.10
Age range, years	32.5–78.4		24.0–82.5		27.1–79.7		
							
*Treatment arm*							
CP	89	51	210	48	185	59^b^	0.48
CPLD	84	49	225	52	129	41^b^	
							
*ECOG* *performance status*							
0	109	63	263	60	199	64	0.69
1	55	32	156	36	92	30	
2	6	3	10	2	9	3	
Ovary as primary site of disease	153	88	399	92	268	86^b^	0.21
Serous subtype	128	74	305	70	227	73	0.61
							
*Histologic grade*							
1	5	3	23	5	24	8	0.21
2	38	22	113	26	67	22	
3	108	62	236	54	152	49	
							
*FIGO staging*							
I or II	23	13	46	11	46	15	0.76
III or IV	145	84	381	88	255	82	
Measurable disease	108	62	238	55	221	71^b^	0.10
>1 sites of metastatic disease	94	54	212	49	176	57	0.30
Tumour size ⩾5 cm	32	19	71	16	69	22	0.82
Baseline CA125⩾100 IU	122	72	270	63	214	69	0.08
							
*Interval since last chemotherapy*							
6–12 months	55	32	149	34	122	39	0.47
>12 months	118	68	286	66	189	61	
							
*No. of previous lines of chemotherapy*							
1	149	86	372	86	262	84	0.37
2	24	14	61	14	49	16	
Surgery for this relapse	33	19	109	25	35	11^b^	0.40
Baseline white blood cell count >6.0 × 10^9^ per litre	144	83	220	51	223	72^b^	<0.0001

Abbreviations: CA=cancer antigen; CP=carboplatin–paclitaxel; CPLD=carboplatin–liposomal doxorubicin; ECOG=Eastern Cooperative Oncology Group; FIGO=Federation of Gynecology and Obstetrics.

a For comparison between baseline characteristics of patients with and without leukopenia.

b Statistically significant difference (*P*<0.05) in baseline characteristics of patients with and without nadir blood count.
